# Mapping adsorption on ionic surfaces via a pairwise potential-based high-throughput approach

**DOI:** 10.1107/S1600576725005230

**Published:** 2025-07-16

**Authors:** Eric Mates-Torres, Piero Ugliengo, Albert Rimola

**Affiliations:** a Departament de Química Universitat Autònoma de Barcelona Campus de la UAB 08193Bellaterra Catalonia Spain; b Dipartimento di Chimica and Centre for Nanomaterials for Industry and Sustainability (NIS) Università degli Studi di Torino via P. Giuria 7 TorinoI-10125 Italy; Shiv Nadar Institution of Eminence, India

**Keywords:** automation, interactions, potential energies, high-throughput techniques, surfaces

## Abstract

Understanding molecular adsorption on ionic surfaces is crucial for a variety of chemical applications, from heterogeneous catalysis to prebiotic chemistry. Traditional approaches for identifying adsorption sites often rely on computationally expensive methods such as density functional theory (DFT), which limits their applicability to chemically complex surfaces. In this work, we propose an automated high-throughput approach to obtain a complete picture of the adsorbate–surface interaction by means of pairwise Coulomb and Lennard–Jones potentials. Using a grid-based surface scan to calculate per-site potential energies of adsorption, this method efficiently predicts global adsorption minima and all potential binding modes of a surface–adsorbate system, with the only user input being the surface CIF. Our approach is validated by studying formaldehyde (H_2_CO) adsorption on forsterite (Mg_2_SiO_4_), a common silicate, and l-cysteine adsorption on cadmium sulfide (CdS). The predicted adsorption configurations and energies are compared with DFT values in the literature, showing good agreement and confirming the accuracy of our method. Our workflow provides a rapid means of exploring large configurational spaces and identifying stable adsorption structures, making it particularly useful for complex surfaces with multiple interaction sites. The simplicity of the model, combined with its accuracy, suggest it could be employed to discover new catalytic pathways on chemically complex ionic surfaces.

## Abstract 2

This work presents a high-throughput method for predicting molecular adsorption sites on ionic surfaces using pairwise Coulomb and Lennard–Jones potentials. Requiring only a CIF as input, the approach enables rapid identification of stable adsorption structures, offering an efficient pathway for studying complex surface–adsorbate interactions.

## 1. Introduction

Understanding the interface between molecules and solid-state surfaces is paramount to unravel a myriad of chemical processes, and considerable efforts have been dedicated to quantifying the underlying interactions using quantum mechanical methods on a wide range of applications. For instance, thorough molecular adsorption studies have been performed to tune and enhance the activity and selectivity of heterogeneous catalysts for energy applications (Sahm *et al.*, 2021 ▸; Sahm *et al.*, 2022 ▸; Marciniak *et al.*, 2020 ▸) or to decipher how minerals may have driven the formation of relevant biomolecules from prebiotic species (Rimola *et al.*, 2013 ▸; Perrero *et al.*, 2023 ▸; Bancone *et al.*, 2023 ▸; Vinogradoff *et al.*, 2024 ▸). However, accurately determining *where* and *how* molecules adsorb (and potentially react) on a given surface is as relevant as it is challenging, since a surface may contain multiple sites with vastly different affinities towards adsorbed molecular species and/or reaction intermediates (Vogt & Weckhuysen, 2022 ▸). This was promptly addressed in the fields of homogeneous catalyst design and drug discovery. Sampling methods, wrapped around the semi-empirical GFN*n*-xTB methods, have been proposed to provide a complete screening of the chemical space governing the formation of inter- and intramolecular interactions (Pracht *et al.*, 2020 ▸; Pracht *et al.*, 2024 ▸; Iribarren & Trujillo, 2022 ▸), while score-based docking strategies allow the determination of the interactions of a target molecule within the binding site of a protein or, more recently, of any molecular cluster (Pagadala *et al.*, 2017 ▸; Verdonk *et al.*, 2003 ▸; Plett & Grimme, 2023 ▸). In the case of surface–adsorbate interfaces, molecular dynamics simulations and umbrella sampling strategies have been used to explore their large chemical space with the target of locating a global minimum (Singh & Sharma, 2022 ▸; Wei *et al.*, 2018 ▸); however, if one aims to obtain a complete description of the interaction between a surface and an adsorbate, high-throughput automated approaches are required.

Recently, many efforts have been devoted to determining potential adsorption sites on metal surfaces in a high-throughput fashion, given their inherently simple structure and their broad use in catalysis. For instance, the periodic and highly ordered arrangement of metal surface atoms can be used in the identification of potential surface sites using a simple triangulation algorithm as a basis for high-fidelity density functional theory (DFT) calculations, as devised by Montoya & Persson (2017 ▸). This principle has been implemented in tools such as the *Xsorb* library (Pedretti *et al.*, 2023 ▸) and the *Alloy Catalysis Automated Toolkit* (*ACAT*) package (Han *et al.*, 2023 ▸). The latter contains a set of pre-defined adsorption sites on metals and metal oxides onto which a user-defined surface can be mapped, providing a database of initial adsorption guesses that can be optimized using either DFT or semi-empirical methods. Recently, *ACAT* was used by Johnson *et al.* (2023 ▸) to generate a database of adsorbates and saddle-point guesses at the semi-empirical GFN1-xTB level to perform an adsorption screening and describe the kinetics of Cu-promoted reaction mechanisms, as implemented in the *Pynta* workflow. Other tools have also been developed to generate adsorption complex candidates automatically for their use in DFT calculations, such as the *Automatic Surface Adsorbate Structure Provider* (*ASAP*) algorithm (Wilson & Muhich, 2022 ▸) and the *Surfkit* toolkit (Liu & Zhang, 2024 ▸). Other open-source tools such as the *DockOnSurf* package (Martí *et al.*, 2021 ▸) allow for the exploration of the adsorption modes of flexible ligands onto metal surfaces given a set of anchor points, albeit in a serial fashion, yielding potential candidates for further analysis using quantum mechanical methods.

Automation strategies like those discussed here have allowed for the rapid development of semi-empirical- and DFT-based databases, which have been used to train machine-learning models and force fields with huge potential for discovering novel catalysts for energy applications (Chanussot *et al.*, 2021 ▸; Pablo-García *et al.*, 2023 ▸).

Despite the advances in automation strategies that have arisen in the past five years, most approaches in the literature have two major drawbacks: (i) the surface is treated as a passive actor in the adsorption process, where selection of the most stable adsorption arises from user-defined initial configurations that may show bias towards a given metastable configuration within the complex potential energy landscape, or (ii) structure post-processing still requires the use of more advanced quantum mechanical methods, hindering the applicability of the workflows for large and chemically complex systems. Addressing these issues is more urgent in the case of other more complex surfaces such as those formed by ionic compounds, which comprise a wide variety of Earth-abundant structures with vastly different atom distributions on their surfaces, often displaying convoluted patterns with steps and kinks (particularly in the case of ternary oxides such as silicates or carbonates). Correctly identifying molecular adsorption sites on these surfaces is crucial. For instance, the exposed negatively and positively charged surface atoms on ionic surfaces may induce combined Lewis acid and basic effects with incoming molecules, promoting charge delocal­ization and inducing changes in their dipoles which dramatically influence molecular reactivity (Vinogradoff *et al.*, 2024 ▸). In these cases, geometry-guided workflows to identify potential adsorption sites, such as triangulations based on the positions of the surface atoms, may potentially generate an unreasonable number of structures; these strategies, when considering the conformational complexity of larger adsorbates, result in a combinatorial diversity of adsorbates at chemically distinct sites, rendering these approaches applicable to simple mol­ecules only if coupled with quantum chemical methods (Mates-Torres & Rimola, 2024 ▸).

**Figure 1:**
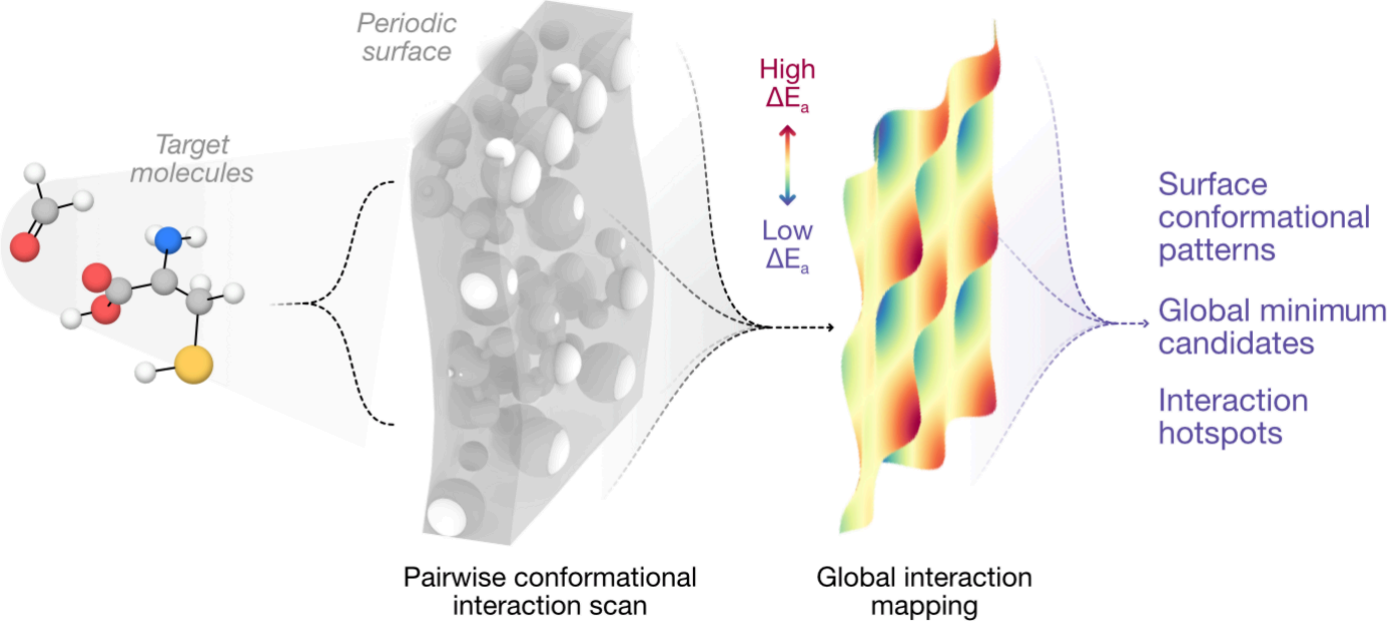
Diagram of the high-throughput approach for multi-conformational adsorption analysis on a solid surface. A global interaction map based on a pairwise interaction scheme, which includes parameterized Lennard–Jones and electrostatic contributions, allows for the determination of the intersection patterns on the surface, from which global minima candidates and various conformational regions can be extracted.

To overcome the limitations of current quantum chemical methods for the analysis of large numbers of chemical structures in adsorption studies, classical pairwise potentials such as interatomic Coulomb and Lennard–Jones interactions have historically been used to model the interactions between non-reactive species, especially in biochemistry, as they offer an accurate compromise between speed and chemical accuracy (Brooks *et al.*, 2009 ▸; Harrison *et al.*, 2018 ▸). These potentials have been previously used for the quantitative modelling and classification of subsets of adsorption species of simple molecules on oxides with remarkable accuracy (Herbers *et al.*, 2011 ▸; Johnston *et al.*, 2012 ▸). Notwithstanding the relevance of current automation models, in this article we envision a simple strategy of using a coarse-grain potential, such as the ones above, as the basis of an automation model to guide adsorption discovery. If coupled with chemical databases such as PubChem (Kim *et al.*, 2016 ▸), we demonstrate that a grid-based surface scan using a pairwise potential consisting of parameterized Coulomb and Lennard–Jones interactions, as schematized in Fig. 1 ▸ and discussed in the *Methods*[Sec sec2] section, yields structures with remarkable similarity to those found manually by means of DFT or using semi-empirical-based automated workflows. In addition to giving key information regarding the most stable candidate adsorption on a chemically complex surface, we also discuss the relevance of this method to unravelling the complete interaction landscape of a molecule and a surface, and its implications for the discovery of novel catalytic pathways.

To investigate the validity of our model to predict global adsorption minima on complex surfaces, we first tackled how simple organic molecules interact with a common silicate, forsterite (Mg_2_SiO_4_); unravelling this interaction is key to understanding how silicates may have promoted the formation of sugars, amino acids and nucleobases in an early Earth under primitive conditions (Vinogradoff *et al.*, 2024 ▸; Campisi *et al.*, 2021 ▸; Vinogradoff *et al.*, 2020 ▸). In particular, we focused our attention on formaldehyde (H_2_CO), which is the precursor for the formation of sugars and amino acids through the formose reaction and the Strecker synthesis, respectively. This interaction has been investigated previously in a report by some of us using an automated approach by means of semi-empirical calculations on sites found by a Delaunay triangulation (Mates-Torres & Rimola, 2024 ▸), which will be the basis for our comparison. Then, the ability of our model to determine correctly not only the global adsorption minima but also the binding mode was also investigated. For this, we turned our attention to amino acids such as l-cysteine (l-cys), whose interaction with ionic surfaces through its carboxyl, amino and thiol groups in a multidentate fashion has been shown to alter the optical properties of fluorescent cadmium sulfide (CdS)-coated quantum dots (QDs), as revealed by thorough DFT calculations (Kuznetsova *et al.*, 2019 ▸).

## 2. Methods

### 2.1. Generation of adsorbate–surface complexes

The CIFs of all optimized surfaces reported in this work were taken from their original reports: Mg_2_SiO_4_ (102) [in the *Pnma* space group setting, corresponding to (120) in the *Pbnm* setting] and CdS (0001) were obtained from Mates-Torres & Rimola (2024 ▸) and Kuznetsova *et al.* (2019 ▸), respectively. Adsorption studies were performed on the *p*(2×1) and *p*(3×3) supercells of Mg_2_SiO_4_ and CdS, respectively, to avoid spurious adsorbate–adsorbate lateral interactions between neighbouring images. In all instances, determination of surface adsorption sites was performed using a grid-based approach, wherein potential adsorption sites were placed equidistantly along the **a** and **b** lattice vectors. Subsequently, target adsorbates were positioned above each surface adsorption site with respect to their centre of mass and rotated along the φ, θ and ψ Euler angles with an angle step of 20°, yielding a total of 4913 distinct starting structures per site. Each of these rotated adsorbates was sequentially approached towards the surface 0.5 Å at a time, building a connectivity matrix at every step using the NeighborList class included in the *Atomic Simulation Environment* (*ASE*) package (Hjorth Larsen *et al.*, 2017 ▸). For efficiency, the connectivity matrix considered only atoms in the adsorbate and on the outermost layer of the surface (or surface atoms), *i.e.* those bulk atoms whose atomic centre did not overlap the radius of any other atom at a higher position on the *z* axis. A divergence in the combined connectivity matrix of the adsorbate–surface complex was deemed to indicate an adsorption–surface interaction, at which point the approach was interrupted. Determination of adsorption structures was parallelized on a site-per-core basis using the *mpi4py* library (Dalcín *et al.*, 2005 ▸).

### 2.2. Tuning of potential-based adsorption energies

The potential adsorption energy *E*_a_ of each adsorbate–surface complex was used to obtain the most stable configuration on each site and to rank the different adsorptions throughout the surface.

On the one hand, we calculated a Coulombic potential *V*_Coulomb_ between all the atoms of the surface’s unit cell and those of the incoming adsorbate,
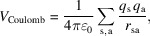
where *r*_sa_ is the distance between the atomic centres of an atom in the adsorbate and one on the surface, *q*_s_ and *q*_a_ are the charges of the atoms on the surface (s) and of the adsorbate (a), respectively, and ɛ_0_ is the vacuum permittivity. In this case, the atomic charges of the atoms on the surface were guessed on the basis of their most probable oxidation state using the Composition class of the *Python Materials Genomics* (*pymatgen*) code (Ong *et al.*, 2013 ▸). Partial atomic charges of the adsorbate molecule were obtained automatically by accessing the adsorbate atomic structure through the PubChem database (Kim *et al.*, 2016 ▸), or by means of single-point calculations using the semi-empirical GFN1-xTB method in cases where the original adsorbate atomic structure is modified.

On the other hand, we also considered a Lennard–Jones potential between surface and adsorbate atoms, *V*_LJ_:
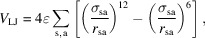
σ is defined as the inter-atomic distance at which the potential becomes zero and is approximated as the sum between the van der Waals radii of particles s and a (Alvarez, 2013 ▸). ɛ, the depth of the potential well, is a measure of the strength of the interaction. *E*_a_ was calculated as the sum of both the Coulombic and Lennard–Jones potentials:
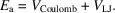


To determine the optimal value for the Lennard–Jones potential well depth, an initial data set of adsorptions of formaldehyde (H_2_CO) on the *p*(2×1) Mg_2_SiO_4_ (102) surface was built with a site grid density with a spacing of 0.7 Å in the *a* and *b* lattice coordinates, yielding a total of 724 sampled adsorption sites. For each atomic configuration, a potential adsorption energy was calculated with a guessed initial ɛ value of 0.005 eV. This value was shown to provide structures with diverse binding modes (not necessarily the most stable ones on each site) and was deemed suitable to represent different interacting scenarios for subsequent benchmarking with more accurate quantum mechanical methods. The studied configurations were ranked according to their *E*_a_ value: at each adsorption site, configurations with lower adsorption energies displayed locally stable adsorptions, while structures with higher adsorption energies corresponded to less stable structures and were thus less representative of the true adsorption modes. In each case, the configuration displaying the lowest adsorption energy on each site was stored into a database. The final configurations were filtered out with an energy-based criterion, where adsorbates with Δ*E*_a_ < 0.1 eV were deemed equivalent due to cell symmetry or close adsorbate proximity. While using a non-optimized value for ɛ is necessary to obtain chemically distinct species for a more representative benchmark, it may underestimate the binding energy of certain configurations and yield structures with spurious interactions as local minima on an adsorption site. Thus, configurations with implausible adsorption modes such as H-down adsorptions were discarded. In total, this approach yielded 66 distinct adsorption configurations, whose binding energy was also computed by means of single-point calculations at the DFT level using the *Vienna Ab-initio Simulation Package* (*VASP*) software (Kresse & Furthmüller, 1996 ▸) with projector-augmented wave (PAW) pseudopotentials (Blöchl, 1994 ▸). In these calculations, the reciprocal space was sampled with a Γ-centred *k*-point grid of 2 × 2 × 1 and a cut-off energy of 500 eV using the Perdew–Burke–Ernzerhof (PBE) functional (Perdew *et al.*, 1996 ▸), matching the methodology found in the baseline literature reports. The DFT-D3 method of Grimme with zero-damping function (Grimme *et al.*, 2010 ▸) was also used to better describe long-range interactions.

The optimal average value of ɛ in the Lennard–Jones potential for the method presented here was determined by plotting the values of *E*_a_ with respect to their corresponding DFT binding energies (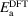). Scatter plots were constructed along different values of ɛ, which was divided by an integer *n* (Fig. S1 in the supporting information). This revealed an optimal value of *n* = 37. Hence, subsequent cohesive energies were calculated with an ɛ coefficient of 1.49 × 10^−4^ eV.

## 3. Results

**Figure 2:**
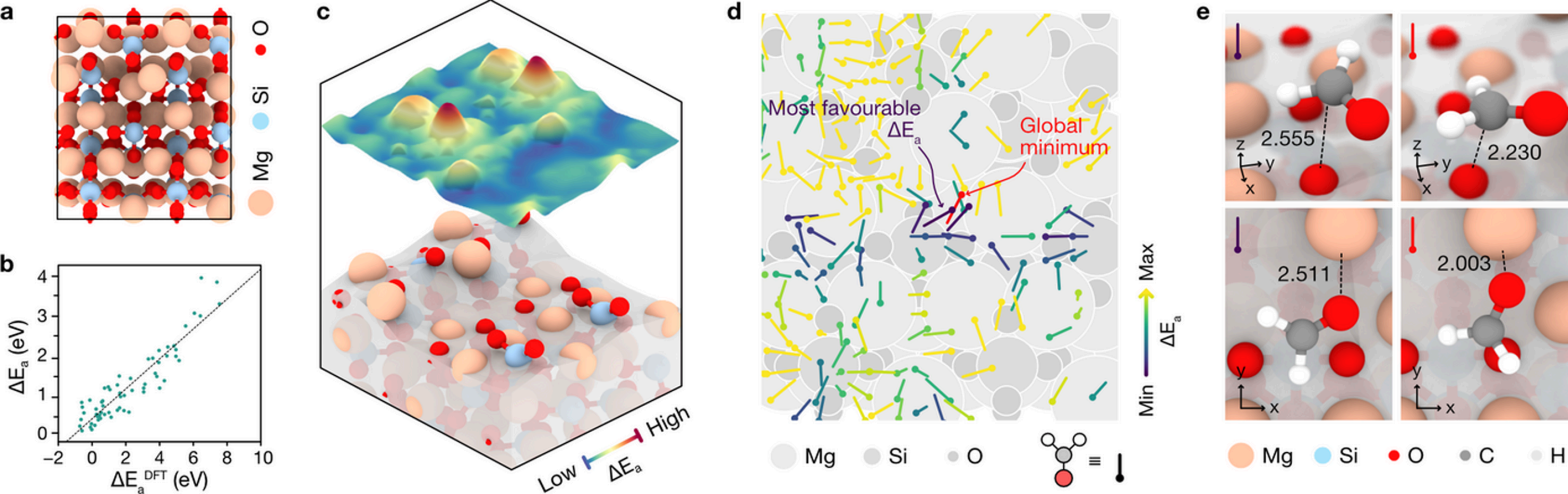
Global screening of the interaction between formaldehyde (H_2_CO) and the *p*(2×1)-(102) surface of forsterite (Mg_2_SiO_4_). (*a*) Top-view representation of the Mg_2_SiO_4_ (102) surface used in this work. (*b*) Scatter plot representation of the calculated adsorption energies (Δ*E*_a_) with respect to the DFT binding energies (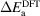) after parametric optimization. (*c*) Heatmap showing the interaction hotspots of formaldehyde on the surface of forsterite; colder and warmer regions depict sites hosting adsorbates with lower and higher adsorption energies, respectively, with the most stable adsorptions within the blue regions and the least stable adsorptions within the red ones. (*d*) Distribution of symmetrically distinct H_2_CO adsorptions on the forsterite surface obtained with the presented workflow. Stable configurations with lower adsorption energies are depicted in blue, while less stable adsorptions with higher adsorption energies are coloured in yellow; the global minimum found through high-throughput semi-empirical-based methods in our baseline study is depicted in red. Surface atoms are greyed out for clarity. (*e*) Side- and top-view representations of the global minima found using our classical-based workflow (left-hand panels) and the DFT-optimized most stable adsorption in the literature (right-hand panels). Relevant interatomic distances are shown in ångströms.

The surface used to address the determination of global adsorption minima, which corresponds to the (102) termination of Mg_2_SiO_4_, is shown in Fig. 2 ▸(*a*). To determine the validity of our adsorption energies based on a pairwise interatomic potential, we applied our algorithm on a coarse grid sampling using H_2_CO as a benchmark adsorbate, and a preliminary set of parameters in the adsorption energy equation, as described in the *Methods*[Sec sec2] section. This yielded a set of theoretically most stable adsorption structures on each site. Far from being the optimal structures, these were representative of the broad range of configurations that H_2_CO may attain on the surface, interacting with the Mg and O surface atoms in a flat conformation or monodentately through its terminal O atom. The pairwise potential-based adsorption energies of the resulting 66 non-equivalent adsorptions were compared with their corresponding 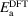, computed *via* single-point calculations. After parametric optimization, the obtained *E*_a_ values were found to be directly proportional to 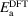, with a linear regression score of 0.85, as shown in Fig. 2 ▸(*b*). The parameterization process reveals no linear correlation between *E*_a_ and 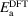 when only Coulombic interactions considered (*i.e.* when ɛ tends to 0); instead, our approach shows that considering the Coulomb potential allows us to discard unsuitable adsorption structures within a given adsorption site when interatomic repulsion with the surface is present, yielding more realistic local minima at each investigated site.

Next, we employed the newly found parameters to determine the relative adsorption of H_2_CO on the Mg_2_SiO_4_ surface. The result from that interaction is represented in Fig. 2 ▸(*c*) as a heatmap on the Mg_2_SiO_4_ surface, showing regions where the adsorption energy is lowest (and thus hosts more stable adsorbates) in blue, while regions with higher adsorption energies, hosting less stable adsorption configurations, are shown in red. While the *p*(2×1) cell multiplicity is made apparent in this representation, this analysis also provides key information on the most active regions for adsorption, as shown in the low-adsorption energy region stretching across the centre of the surface. All the adsorptions found using our workflow are filtered with an energy-based criterion and their top view is displayed in Fig. 2 ▸(*d*), where the least (most) energetic adsorption configurations are coloured in blue (yellow).

To obtain an estimated globally most stable structure for the adsorption of H_2_CO, we proceeded to optimize, at the DFT level, the least energetic adsorption found by a semi-empirical-based sampling of the surface adsorption sites. This method, albeit more resource demanding, was used to ensure an accurate representation of the least energetic adsorption configuration. Our simulations show that the most stable H_2_CO adsorption is depicted by a flat configuration on the surface, with the O termination coordinated with a surface Mg atom at a distance of 2.003 Å and its C atom at a distance of 2.230 Å from a surface O site. The relative position of the DFT-optimized global minimum with respect to the results found using our classical-based workflow, as schematized in Fig. 2 ▸(*d*), reveals that our approach is able to predict the most stable binding region with remarkable accuracy, as revealed by the dark-blue adsorption configurations surrounding the *true* global minimum, represented in red. Side and top views of the lowest-energy, and thus most stable, representation and the global DFT-based adsorption minimum are depicted in the left- and right-hand panels of Fig. 2 ▸(*e*), respectively; this shows the remarkable similarity of our least energetic configuration and the *true* global minimum.

**Figure 3:**
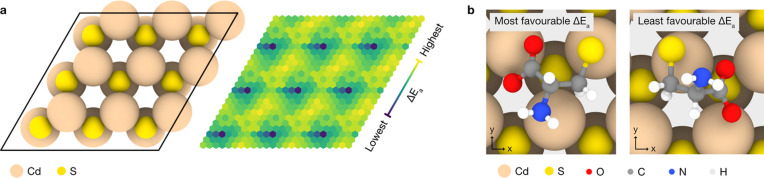
A classic-based global interaction screening of l-cys on the *p*(3×3) CdS (0001) surface reveals a complex range of multi-conformational adsorptions. (*a*) Top view of the CdS (0001) unit cell used to study the adsorption of l-cys (left), along with the global interaction heatmap (right), displaying more stable adsorption conformations in dark blue and less stable adsorptions in yellow. (*b*) Top-view representations of the most stable and least stable conformations on the surface, showcasing the ability of the workflow to correctly identify divergences in adsorption stability driven by sterically driven conformational changes.

The presented methodology can also qualitatively predict the conformational space of a flexible organic molecule upon adsorption on a CdS QD surface. Specifically, l-cys was used to obtain a multi-conformational screening of the surface adsorption sites, where the most stable adsorption in each site corresponded to the candidate with the lowest adsorption energy from all possible configurations contained within the Pubchem database. To simulate better the *operando* pH in experiments, all l-cys structures considered herein were deprotonated in their COOH and SH groups, resulting in molecules with effective charges of −2. Note that, while the reference literature calculations were performed using *VASP*, which includes a background positive charge to avoid spurious net charges in periodic unit cells, the point-charge methodology developed herein (see *Methods*[Sec sec2] section) overcomes this issue and works regardless of the atomic charge of the adsorbate. This yielded a variety of adsorbate conformations throughout the surface, ranging from bidentate adsorption modes through the terminal S^−^ and either NH_2_ or O^−^, in a tridentate fashion through the S^−^, NH_2_ and one carboxyl­ate O^−^ atom, or in a tetradentate mode, in which all terminal species anchor the surface in concert. Both tridentate and tetradentate conformations, which were observed to be the prevalent configurations in low-coverage scenarios in the literature, were found to be omnipresent throughout the surface, with tetradentate conformations governing the most stable adsorption sites on the basis of their adsorption energies [Fig. 3 ▸(*a*)]. Our results point out the necessity for the mol­ecule’s centre of mass to be located at surface hollow positions, so that all terminal atoms of l-cys can coordinate with positively charged surface Cd^2+^ ions, as shown in the left-hand panel of Fig. 3 ▸(*b*). Alternatively, our simulations showed that the most energetic, and thus least stable, structures all corresponded to bidentate adsorption modes coordinating with either one or two surface Cd^2+^ atoms, all lying on the highest adsorption energy regions in the right-hand panel of Fig. 3 ▸(*a*). For illustration purposes, a top-view representation of the least stable structure found with our approach is depicted in the right-hand panel of Fig. 3 ▸(*b*).

## 4. Conclusions

Revealing the complete nature of the interaction between chemically complex ionic surfaces and relevant adsorbates is key for a wide range of applications. In this work, we have discussed the ability of an automated grid-based workflow coupled with a simple pairwise interatomic potential to predict the relative adsorbate–surface interaction strength, adsorbate position and per-site conformation. In our approach, two cases were studied, namely the adsorption of H_2_CO on a common silicate, Mg_2_SiO_4_, and of a relevant amino acid for optical applications, l-cys, on a CdS QD surface. Firstly, our analysis reveals that an adsorption energy based on adsorbate–surface Coulombic and Lennard–Jones potentials is directly proportional to the adsorption energy calculated with high-fidelity DFT calculations. This paves the way for the automated determination of the nature of the interaction of H_2_CO on Mg_2_SiO_4_ using a grid-based approach, yielding an adsorption energy heatmap that shows the most stable adsorption positions on the studied surface. The best candidates (*i.e.* those depicting the lowest adsorption energies) using this method­ology were compared with the most stable structure found *via* more resource-demanding semi-empirical-based automated approaches in the literature, revealing a good agreement between the methods and showing that our classical-based approach can yield accurate candidate adsorption structures for use in adsorption and reactivity studies. Finally, we have demonstrated that coupling this potential-based approach with conformer-containing chemical databases allows for a complete determination of not only the adsorption sites and relative adsorption energies but also their binding mode. Specifically, this approach yields reasonable binding structures of l-cys on a CdS surface, which were shown to be predominantly bound in a tetradentate conformation atop surface hollow sites, in remarkable agreement with the most stable structures found by means of DFT analyses in the literature.

The use of pairwise-based potentials is the basis for a myriad of automated approaches, such as docking algorithms, molecular dynamics simulations and conformational analyses in the fields of materials chemistry and biochemistry. In this work, we have demonstrated that such a method can be applied to obtain a key understanding of the interaction between a chemically complex ionic surface and an adsorbate, at a fraction of the computational cost of quantum-mechanics-based approaches. On the basis of our results, we argue that this method is essential for the directed discovery of novel synthetic pathways catalyzed by ionic structures.

## Supplementary Material

Additional figure. DOI: 10.1107/S1600576725005230/ui5030sup1.pdf

## Data Availability

All computational data reported in this work, including DFT calculations and *ASE* databases arising from this work, as well as the Python script used in our automated approach, can be accessed via an on-line Zenodo repository through the following URL: https://doi.org/10.5281/zenodo.14809725.
