# Supplementary material for: Mapping adsorption on ionic surfaces via a pairwise potential-based high-throughput approach
Source: J Appl Crystallogr. 2025 Jul 16;58(Pt 4):1462–8. doi: 10.1107/S1600576725005230 (PMC12321012; doi:10.1107/S1600576725005230)
Supplement: Supplementary file 1 [file j-58-01462-sup1.pdf]

JOURNAL OF
APPLIED
CRYSTALLOGRAPHY

Volume 58 (2025)

Supporting information for article:

Mapping adsorption on ionic surfaces via a pairwise potential-based high-throughput approach

Eric Mates-Torres, Piero Ugliengo and Albert Rimola

Supporting information

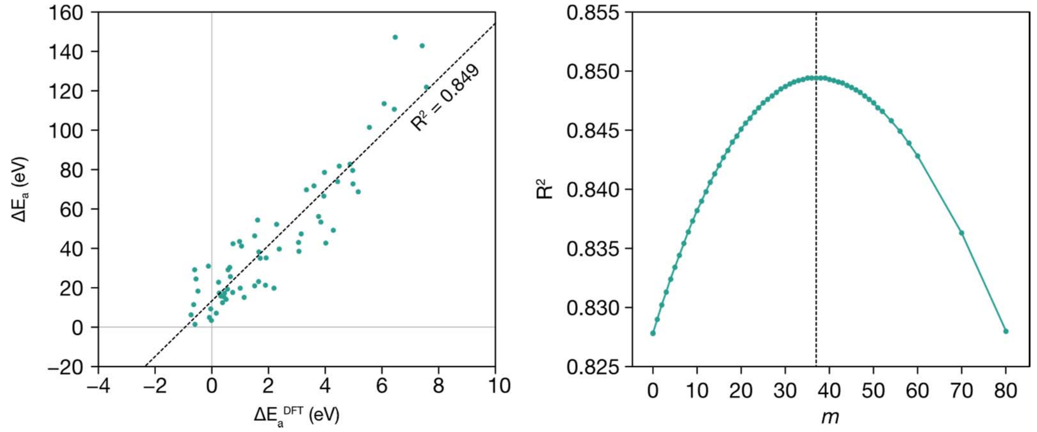

Figure S1. On the left, a scatter plot of the potential adsorption energy of H_2CO on the surface of forsterite found through the presented algorithm (E_a) versus the adsorption energy obtained by means of single-point DFT calculations (E_a^{DFT}), with an ε parameter in the description of V_{LJ} of $1.49 \cdot 10^{-4}$ eV. This was found as the value yielding the best linear regression between E_a and E_a^{DFT}, as displayed in the scatter plot on the right, where R^2 is the regression value and m is a constant modifier of the ε parameter.
